# Down-regulation of the tumor suppressor miR-34a contributes to head and neck cancer by up-regulating the MET oncogene and modulating tumor immune evasion

**DOI:** 10.1186/s13046-021-01865-2

**Published:** 2021-02-17

**Authors:** Xun Wu, Yi-Shing Lisa Cheng, Mathew Matthen, Angela Yoon, Gary K. Schwartz, Shashi Bala, Alison M. Taylor, Fatemeh Momen-Heravi

**Affiliations:** 1 Cancer Biology and Immunology Laboratory, College of Dental Medicine, Columbia University Irving Medical Center, New York, NY USA; 2 Division of Periodontics, Section of Oral, Diagnostic and Rehabilitation Sciences, Columbia University College of Dental Medicine, New York, NY USA; 3 Department of Maxillofacial Surgery, Guangxi Medical University College of Stomatology, Nanning, Guangxi China; 4 Department of Diagnostic Sciences, Texas A&M University College of Dentistry, Dallas, TX USA; 5 Department of Medicine Division of Hematology/Oncology, Columbia University Irving Medical Center, New York, NY USA; 6 Division of Pathology, Columbia University College of Dental Medicine, New York, NY USA; 7 Department of Medicine, University of Massachusetts Medical School, Worcester, MA USA; 8 Herbert Irving Comprehensive Cancer Center, Columbia University Irving Medical Center, New York, NY USA; 9 Department of Pathology and Cell Biology, Vagelos College of Physicians and Surgeons, Columbia University Irving Medical Center, New York, NY USA

**Keywords:** Head and neck cancer, miR-34a, Aneuploidy, P53, MET, micoRNA

## Abstract

**Background:**

MicroRNAs (miRs) have been shown to play an important role in tumorigenesis, including in head and neck squamous cell carcinoma (HNSCC). The miR-34 family is thought to play a role in tumor suppression, but the exact mechanism of their action in HNSCC is not well understood. Moreover, the impact of chromosomal changes and mutation status on miR-34a expression remains unknown.

**Methods:**

Differential expression of miR-34a, MET, and genomic alterations were assessed in the Cancer Genome Atlas (TCGA) datasets as well as in primary HNSCC and adjacent normal tissue. The biological functions of miR-34a in HNSCC were investigated in samples derived from primary human tumors and HNSCC cell lines. The expression of MET was evaluated using immunohistochemistry, and the molecular interaction of miR-34a and MET were demonstrated by RNA pulldown, RNA immunoprecipitation, luciferase reporter assay, and rescue experiments. Lastly, locked nucleic acid (LNA) miRs in mouse xenograft models were used to evaluate the clinical relevance of miR-34a in HNSCC tumor growth and modulation of the tumor microenvironment in vivo.

**Results:**

Chromosome arm 1p loss and P53 mutations are both associated with lower levels of miR-34a. In HNSCC, miR-34a acts as a tumor suppressor and physically interacts with and functionally targets the proto-oncogene MET. Our studies found that miR-34a suppresses HNSCC carcinogenesis, at least in part, by downregulating MET, consequently inhibiting HNSCC proliferation. Consistent with these findings, administration of LNA-miR-34a in an in vivo model of HNSCC leads to diminished HNSCC cell proliferation and tumor burden in vitro and in vivo, represses expression of genes involved in epithelial-mesenchymal transition, and negates the oncogenic effect of MET in mouse tumors. Consistently, LNA-miR-34a induced a decreased number of immunosuppressive PDL1-expressing tumor-associated macrophages in the tumor microenvironment. In HNSCC patient samples, higher levels of miR-34a are significantly associated with a higher frequency of Th1 cells and CD8 naïve T cells.

**Conclusions:**

Our results demonstrate that miR-34a directly targets MET and maintains anti-tumor immune activity. We propose miR-34a as a potential new therapeutic approach for HNSCC.

**Supplementary Information:**

The online version contains supplementary material available at 10.1186/s13046-021-01865-2.

## Background

MicroRNAs (miR) are evolutionarily conserved non-coding RNAs that play roles in fundamental cellular functions by post-translational suppression of gene expression [1]. In cancer pathogenesis, miRs can exert both anti-tumorigenic and pro-tumorigenic effects by virtue of miR-specific and context-dependent mechanisms. The miR-34 family is considered one of the regulators of tumor suppression [2]. In mammals, the miR-34 family includes three miRs that are encoded by two different genes. miR-34a is encoded by its own transcript, while miR-34b and miR-34c share a common transcript and are dysregulated in some cancers [3]. Decreased levels of miR-34a expression have been reported in and linked to the pathogenesis of numerous types of cancer, including ovarian cancer, colorectal cancer, pediatric neuroblastoma, hepatocellular carcinoma, triple-negative breast cancer, lung adenocarcinomas, bladder cancer, prostate cancer, and osteosarcoma [4–12]. The locus harboring the miR-34a transcripts is in a region associated with a chromosomal fragile site on chromosome 1p. The 1p chromosome arm is frequently deleted in many cancers, including squamous cancers [13].

Recent findings showed that transcription of the miR-34 family is controlled by the tumor suppressor p53 [14–16]. p53 has multiple binding sites in regions proximal to the *MIR34A* promoter [17, 18]. Other gene regulatory mechanisms, such as CpG methylation of the MIR34A promoter, have been reported as the main causes of miR-34a down-regulation [19]. Mechanistic studies demonstrated that miR-34a itself is a key player in the p53 network, mediating the biological function of p53 by regulating the expression of different genes [20]. miR-34a directly suppresses HDM4, a negative regulator of p53, forming a positive feedback loop acting on p53 [21]. Treatment with a miR-34a inhibitor attenuates p53-mediated apoptosis in response to genotoxic stress, whereas the ectopic expression of miR-34a causes a significant reprogramming of gene expression and induces apoptosis and cell cycle arrest [22]. miR-34a has been shown to directly target the 3′ untranslated regions (UTRs) of numerous mRNAs with roles in oncogenesis beyond p53, including Bcl-2, PIK3R2, c-Myc, SIRT1, VAMP2, IKBKE, MYH9, KLRK1, CD11A, SDK4–6, Notch1, TRAFD1, and CCR1 [23–26], which may contribute to its tumor-suppressive function.

In head and neck squamous cell carcinoma (HNSCC), miRs can serve as biomarkers for diagnosis and prognosis [27, 28]. Reduction of miR-34a was detected in HNSCC cell lines and tumor tissues and was associated with cell proliferation and angiogenesis [29]. However, the genetic alterations and molecular network that cause miR-34a downregulation in HNSCC are not well understood. Moreover, the mechanistic role of miR-34a downregulation in the pathogenesis of HNSCC and the tumor microenvironment is not well established.

Our bioinformatics and experimental analyses identified several genes, including the MET proto-oncogene, that are directly regulated by miR-34a. This regulation has implications for the role of miR-34a in suppressing tumor growth and modulating the tumor microenvironment. MET is a receptor tyrosine kinase [30], deregulated in many types of human malignancies including breast cancer, lung cancer, bladder cancer, hepatocellular carcinoma, and melanoma [31, 32]. Although abnormal activation of MET in some cancers, such as hepatocellular carcinoma, is known to be correlated with poor prognosis [33], the role of the miR-34a-MET axis in HNSCC has not been investigated. Additionally, the role of miR-34a in the tumor microenvironment in HNSCC is also yet to be elucidated. miR-34a based therapeutics have been brought to melanoma clinical trials as a first-in-class miR therapy (https://clinicaltrials.gov/ct2/show/NCT02862145) [34], but this has not been explored in HNSCC. In the present study, we determined that miR-34a suppresses HNSCC growth by inducing cell cycle arrest and senescence, and we identified an anti-tumor miR-34a regulatory function in HNSCC and tumor-associated macrophages (TAMs) in vivo.

## Methods

### TCGA samples

Our analysis included the TCGA pan-cancer atlas dataset, which includes 10,522 tumors across 33 cancer types including 528 HNSCC samples. The following data is available at gdc.cancer.gov/node/977: cancer type, p53 mutation status, chromosome arm aneuploidy status, miR-34a expression, and mRNA gene expression. Only samples with both miR expression profiling and mutation or mRNA expression profiling were considered. Correlation between miR-34a and MET mRNA was quantified with Pearson’s correlation coefficient, and correlation coefficients with Bonferroni-corrected *p*-value ≤0.05 were considered statistically significant. The Mann-Whitney U test was used to compare expression between different groups of samples. We generated survival curves of HNSCC cases in the TCGA- cohort according to the expression status of the MET gene stratified based on HPV status. A group cutoff of “quartile” was identified, and the Kaplan–Meier curve was plotted. Univariate and multivariable Cox proportional hazards regression was used to assess association with overall survival controlling for different covariates using R software. Estimation of individual immune subtype fractions by xCell in TCGA samples is publicly available at xcell.ucsf.edu.

### Gene set enrichment analysis (GSEA)

First, within the HNSCC samples of the TCGA, we performed Pearson correlations to identify genes whose expression correlates with miR-34a expression. Genes that correlated with miR-34a expression with a Bonferroni corrected *p*-value of < 0.01 were analyzed by the GSEA pre-ranked algorithm to look for enrichment of these genes in the GSEA hallmark datasets.

### Clinical samples

Clinical samples and plasma were obtained from the Tumor Bank of Columbia University Irving Medical Center, Biomarker Core of Herbert Irving Comprehensive Cancer Center, and University of Massachusetts Medical School Conquering Diseases Biorepository. All samples were collected based on an institutional review board guideline. Forty-two histologically confirmed HNSCC samples from a Columbia HNSCC cohort were included in this study, with 80% tumor contact in each sample. The cohort demographics were the following: 26 males, 16 females; age, mean standard deviation (SD) of 64 (6.3) years. 55% of patients were stage I and II, and 45% were stage III and IV HNSCC. Plasma samples were obtained from 14 HNSCC patients and 14 age- and sex-matched healthy controls. The mean (SD) of the age of HNSCC was 62 (5.2) years, and the mean (SD) age of healthy controls was 60 (6.1). There were five females and nine males in each group.

### In vitro pull-down assay

miR-34a-5p biotin labeled probe was synthesized by IDT with the probe sequence of 5’Biotin-TGG CAG TGT CTT AGC TGG TTG T as well as the negative control probe with the sequence of 5’Biotin-ACG TGA CAC GTT CGG AGA ATT. RNA samples isolated from CAL27 cells using TRI reagent (Zymo) and treated with DNase I according to the manufacturer’s instructions. Biotin-labeled miR-34a-5p pull-down probe or a negative control probes in the final volume of 100ul and concentration of 1, 0.5, or 0.25 uM were used. 20 μg of RNA was incubated with probe for 1 h at 4 °C. A μMACS separator (Miltney) was used for purification following the manufacturer’s protocol. The levels of pulled-down MET were quantified by a real-time RT-PCR assay and normalized to the total input.

### Absolute quantification of miR-34a-5p and miR-34a-3p

Synthetic cel-miR-39, miR-34a-5p mimic, and miR-34a-3p mimic (Applied Biosystem) were serially diluted to final concentrations of 300 nM, 30 nM, 3 nM, 0.3 nM, 0.03 nM, 3 pM, 0.3 pM, 0.03 pM, 3 fM, and 0.3 fM. miR-34a-5p, miR-34a-3p, and cel-miR-39 serial dilutions were reverse-transcribed and assayed using real-time PCR analysis concurrently with RNA extracted from tumor samples and normal tissue. 2.5 μL of exogenous cel-miR-39 at a concentration of 200 amol/μl was introduced as an exogenous normalizer before RNA extraction in all samples. Standard curves for miR-34a-5p and miR-34a-3p and cel-miR-39 were included on each plate of the TaqMan microRNA assays to convert the Ct values of each sample into the corresponding number of microRNA copies. The absolute quantification result of miR-34a-5p and miR-34a-3p was obtained by normalization to cel-miR-39.

### Tumor model

All experimental procedures were approved by Institutional Animal Care and Use Committees. Six-week old female nude mice (NU/J) were purchased from Jackson Laboratories. The animals were subjected to a 12 h light cycle, relative humidity of 55%, and temperature of 21 ± 2 °C. After one week of adaptation, animals were inoculated with HTB-43 tumor cells (2 × 10^6^ cells in 100ul volume of PBS) in tongue. HTB-43 xenograft model induces aggressive HNSCC tumors [35]. Tumor volume was measured continuously from day 4 post implant to following the development of tumors. Ten days post implantation of tumor cells, 24 mice were randomized to three group of control (PBS injection), LNA- miR-34a-5p or LNA-miR-34a-5p (*n* = 8 per group; *n* = 24 total). Mice were dosed with LNA- control mimic or LNA-miR-34-mimic (20 mg/kg/ intraperitoneal (i.p)) every week for two weeks at day 10 post tumor injection. Tumor volume measurements were quantified by digital calipers and calculated using the formula (π)/6 × (large diameter) × (small diameter)^2^. The mice were anesthetized by CO2 chamber (70% CO2/30% O2) and cervical dislocation. No adverse events and change in the mice well-being were observed in any of the treatment groups. Tumors were retrieved from animals and went through tumor dissociation protocol for flow cytometry analysis or single cell isolation using a gentleMACS™ Tissue dissociator (Miltenyi) and tumor dissociation kit (Miltenyi) as recommended by the manufacturer. Anti-EpCAM magnetic beads (Miltenyi) were used for isolation of squamous epithelial tumor cells. CD11b magnetic beads (Miltenyi) were used for isolation of TAMs after tumor dissociation as recommended by the manufacturer. Sections of tumors were kept in RNAlater® for RNA isolation or stored at − 80 °C for protein analysis.

### Plasmid construction and luciferase reporter assay

The 3′-untranslated region (UTR) of human MET and PDL1 were amplified from human genomic DNA and individually inserted into the pmirGLO Dual-Luciferase miRNA Target Expression Vector. Mutant PDL1 and MET 3′-UTRs were generated with the QuickchangeXL mutagenesis kit (Stratagene, United States) to disrupt the binding of miR-34a. The fragment of MET and PDL1 3′-UTR mutant was inserted into the pmirGLO Dual-Luciferase miR Target Expression Vector. CAL27 cells were co-transfected with wild-type or mutant MET reporter plasmid and miR-34a-5p mimic or negative control using Lipofectamine 2000 (Invitrogen). Similar approach was taken to verify interaction of miR-34a-5p with PDL1 3’UTR in RAW macrophages. Luciferase activity was measured forty-eight hours post-transfection using Dual-Glo Luciferase Reporter System according to the manufacturer’s instructions (Promega) using a LB96V luminometer (Berthold). Firefly luciferase units were normalized against Renilla luciferase units to control for transfection efficiency. Luciferase activity was averaged from at least 5 replicates.

### Immunohistochemistry

Paraffin-embedded human-epithelial tissues, HNSCC tissues, and dysplastic oral squamous cell tissues were immunostained for MET protein using a laboratory established protocol. Briefly, deparaffinization, sequential ethanol treatment and antigen retrieval was performed. The process was followed by blocking and inactivating endogenous peroxidase with 3% H_2_O_2_, addition of the primary antibody (overnight; 4 °C) and addition of biotin-labeled secondary antibody (Room temperature; 30 mins). DAB was used for staining. Slides were digitally imaged at 20X magnification and analyzed within the Aperio Spectrum Database.

### Western blot

Western blots were performed using the following established laboratory protocols. Cells were lysed in RIPA buffer and protein was quantified using a Bradford assay (Thermofisher). Proteins were separated using 10% SDS-PAGE electrophoresis and transferred onto polyvinylidene difluoride (PVDF) membranes. The membranes were blocked with 5% nonfat milk and incubated at 4 °C overnight with MET, Vimentin, CDH1, GAPDH, and Actin antibodies (1:1000). The membranes were then re-incubated with anti-rabbit (1:5000) secondary peroxidase-labeled antibodies at room temperature for 2 h. The blots were visualized with ECL Plus reagent (Bio-Rad).

### Exosome isolation

Exosomes were extracted from plasma using an established protocol. Plasma samples were centrifuged at 1500 g for 10 min at 4 °C to remove the cellular debris and then at 10,000 g for 20 min to remove the large vesicles. The supernatant was collected and incubated with ExoQuick™ overnight at 4 °C. The mixture was centrifuged twice at 1500 g for 30 min to pellet the exosomes. The pellet was finally resuspended in 200 μl of PBS and used for RNA isolation. For the isolation of exosomes from THP-1 culture medium, supernatants were centrifuged at 700 g for 15 mins to deplete cells and then at 12,000 g for 30 mins to eliminate residual cellular debris, as described previously. The resulting supernatant was passed through 0.4 μm and 0.22 μm filters and concentrated using the Amicon Ultra-15 Centrifugal Filter Unit with Ultracel-100 membrane (Millipore).

### RNA isolation

RNA from cells, exosomes, and plasma was isolated using a QIAzol Lysis reagent and total RNA was isolated using Direct-zol™ RNA MiniPrep isolation kit (Zymo Research). 100 μL of exosome suspension or plasma from plasma of patients with HNSCC or healthy subjects were mixed with 300 μL QIAzol lysis buffer, and the mixture was processed according to the standard protocol. Quantity and quality of the RNA were determined by NanoDrop 1000 (260/280 and 260/230 ratios).

### Quantitative reverse transcription PCR (qRT-PCR)

Quantitative Reverse Transcription PCR (qRT-PCR) was used to determine the expression levels of mRNAs. For mRNA analyses, cDNA was transcribed from 1 μg of total RNA utilizing iScript™ cDNA synthesis kit (Bio-Rad). Real-time quantitative PCR was performed with iTaq Universal SYBR Green Supermix (Bio-Rad). The primer sequences were as follows: human MET (forward), 5′-GAG GCA GTG CAG CAT GTA GT-3′, human MET (reverse), 5′-GAT GAT TCC CTC GGT CAG AA-3′, GAPDH (forward), 5′-TCA GTG GTG GAC CTG ACC TG-3′, GAPDH (reverse), 5′-TGC TGT AGC CAA ATT CGT TG-3′. mouse MET (forward), 5′-GACTTCAGCCATCCCAATGT-3′, mouse MET (reverse), 5′-GGTGAACTTCTGCGTTTGC-3′. human Vimentin (forward), 5′-TGTCCAAATCGATGTGGATGTTTC-3′, human Vimentin (reverse), 5′-TTGTACCATTCTTCTGCCTCCTG-3′. human CDH1 (forward), 5′-GCCTCCTGAAAAGAGAGTGGAAG-3′, human CDH1(reverse), 5′-TGGCAGTGTCTCTCCAAATCCG-3′, mouse Vimentin (forward) 5′-CCCTCACCTGTGAAGTGGAT-3′, mouse Vimentin (reverse) 5′-TCCAGCAGCTTCCTGTAGGT − 3′. mRNA relative levels were calculated using the ΔΔCt method. The relative expression level of each mRNA was presented by 2^–ΔΔCt^.

### microRNA analysis

TaqMan microRNA Assays (Applied Biosystems) was used for detection of miR-34a-3p and miR-34a-5p expression according to manufacturer’s protocol. Briefly, reverse transcription (30 min, 16 °C; 30 min, 42 °C; 5 min 85 °C) was performed using a TaqMan stem loop primer, 15 ng RNA, TaqMan primers and miR reverse transcription kit (Applied Biosystems). qRT- PCR was performed using the TaqMan Universal PCR Master Mix according to the manufacturer’s protocol. RNU-48 was used to normalize the Ct values between the samples. In experiments involving miR analysis of exosomes or plasma, synthetic *C. elegans* (cel)-miR-39 was spiked during the total RNA isolation process and used as normalizer. All experiments were performed in triplicate. miR levels were normalized and the relative expression levels of specific miR were presented by 2^–ΔΔCt^.

### MTT assay

CAL27 or HTB-43 cells were plated in 96-well plates. After 24 h, transfection with 25 nM of miR-34a-5p mimic or control mimic (Ambion) was performed with Lipofectamine RNAiMAX (Thermofisher). 3-(4,5-Dimethylthiazol-2-yl)-2,5-diphenyltetrazolium bromide assay was performed using the Vybrant® MTT Cell Proliferation Assay Kit, as described by the manufacturer. The absorbance of the samples was measured at 595 nm using a microtiter plate reader. Experiments were assayed in triplicate.

### Apoptosis detection assay

Apoptosis levels were measured using an Annexin A5 apoptosis detection Kit (BioLegend) according to the manufacturer’s protocol. HTB-43 and CAL27 cells were pretreated with miR-34a-5p mimic using electroporation as described by our group previously [36]. Cells were collected after 48 h and the treated cells were washed twice with cold BioLegend’s Cell Staining Buffer and then resuspended in Annexin V Binding Buffer at a concentration of 0.25–1.0 × 10 cells/mL. After incubating for 15 min (25 °C), the cells were subjected to flow cytometry analysis to detect the apoptosis.

### Antibodies and reagents

The following primary antibodies were used for this study: MET monoclonal antibody (3D4) (37–0100; Invitrogen); Ago2 Monoclonal Antibody (MA5–23515, Invitrogen); mouse IgG (sc-2025; Santa Cruz); FITC anti-STAT3 Phospho (Tyr705) (Biolegend); anti-mouse CD45 Antibody (30-F11, Biolegend); anti mouse MET antibody (ab51067, Abcam); anti-mouse CD11b Antibody (M1/70, Invitrogen); anti-mouse F4/80 Antibody (BM8; Biolegend); anti-mouse lineage cocktail (145-2C11; RB6-8C5; RA3-6B2; Ter-119; M1/70; Biolegend) and anti-mouse CD274 (PDL1) (10F.9G2; Biolegend), and anti-mouse Vimentin APC-conjugated Antibody (IC2105A, &D). hsa-miR-34a-5p mimic and inhibitors were obtained from Ambion (Thermofisher). pLL3.7 was a gift from Luk Parijs (Addgene plasmid # 11795). pLL3.7 miR-34a-5p was a gift from Judy Lieberman (Addgene plasmid # 25791). The MET vector used in this study included both coding sequence and 3’UTR of MET and was constructed in a pBABE vector. LNA-miR-34a-5p miR was provided by Exiqon. The Lipofectamine RNAiMAX (Invitrogen) or Lipofectamine 2000 (Invitrogen) were used for transfection experiments.

### Cell lines

CAL27 (CRL-2095) was obtained from ATCC and maintained in Dulbecco’s minimal essential medium (DMEM). HTB-43 was obtained from ATCC and maintained in ATCC-formulated Eagle’s Minimum Essential Medium (EMEM). HCSS-4 (CRL-1582) was obtained from ATCC and maintained in ATCC-formulated RPMI-1640 Medium. THP-1 (TIB-202™) was obtained from ATCC and maintained in ATCC-formulated RPMI. RAW 264.7 macrophages (ATCC TIB-71) were obtained from ATCC and cultured in DMEM. All cells were supplemented with 10% fetal bovine serum, 100 U/mL penicillin and 100 mg/mL streptomycin and incubated at 37 °C in 5% CO_2_.

### RNA immunoprecipitation (RIP)- qPCR (RIP-qPCR)

Cells were cross-linked and lysed in IP buffer supplemented with phosphatase/protease inhibitors and RNase inhibitor. Cell lysates were sonicated and 100 μg of total protein was incubated with Ago2 antibody or mouse IgG (non-specific control), the lysates were incubated overnight with either 10μg/ml of ChIP-grade anti-Ago2 or mouse IgG in 4 °C for 90 min, followed by addition of Protein A/G PLUS-Agarose beads and incubation for 80 min. After washing, the eluted RNA samples were further purified with TRI reagent and subjected to TaqMan MicroRNA Assay and qRT-PCR analysis to detect miR-34a-5p and MET, respectively. Data was normalized based on the total input.

### Flow cytometry

A flow cytometry panel consisting of Lin-1, CD45, CD11b, F4/80, and CD274 (PDL1) was used for quantification and characterization of TAMs. Tumor cells were dissociated using a gentleMACS™ Tissue dissociator (Miltenyi) and tumor dissociation kit (Miltenyi) as recommended by the manufacturer. For intracellular staining of Vimentin and phospho-STAT3 (p-STAT3), cells were prepared using the Fix and Perm Kit as recommended by the manufacturer (Invitrogen). Antibody-capture beads (CompBeads, BD Biosciences) were used as single-color compensation controls. Cytometer calibration was performed daily by the use of rainbow fluorescent particles (BD Biosciences), after acquiring unstained and single-color control samples to calculate the compensation matrix. Data were analyzed using FCS Express software.

### Statistical analysis

Student t-tests were performed to compare miR-34a expression level between classes in TCGA data, with *P* < 0.05 considered significant. To identify mRNA expression patterns that significantly correlate with miR-34 expression, a Pearson’s correlation was performed, and *p*-values were corrected using false discovery rate (FDR) and Bonferroni methods. For experimental data, Parametric Student’s t test or Mann-Whitney U test were performed for comparing two groups. Data are demonstrated as mean ± standard error of mean (SEM) or standard deviation (SD). *P* values less than 0.05 were considered as statistically significant.

## Results

**Fig. 1:**
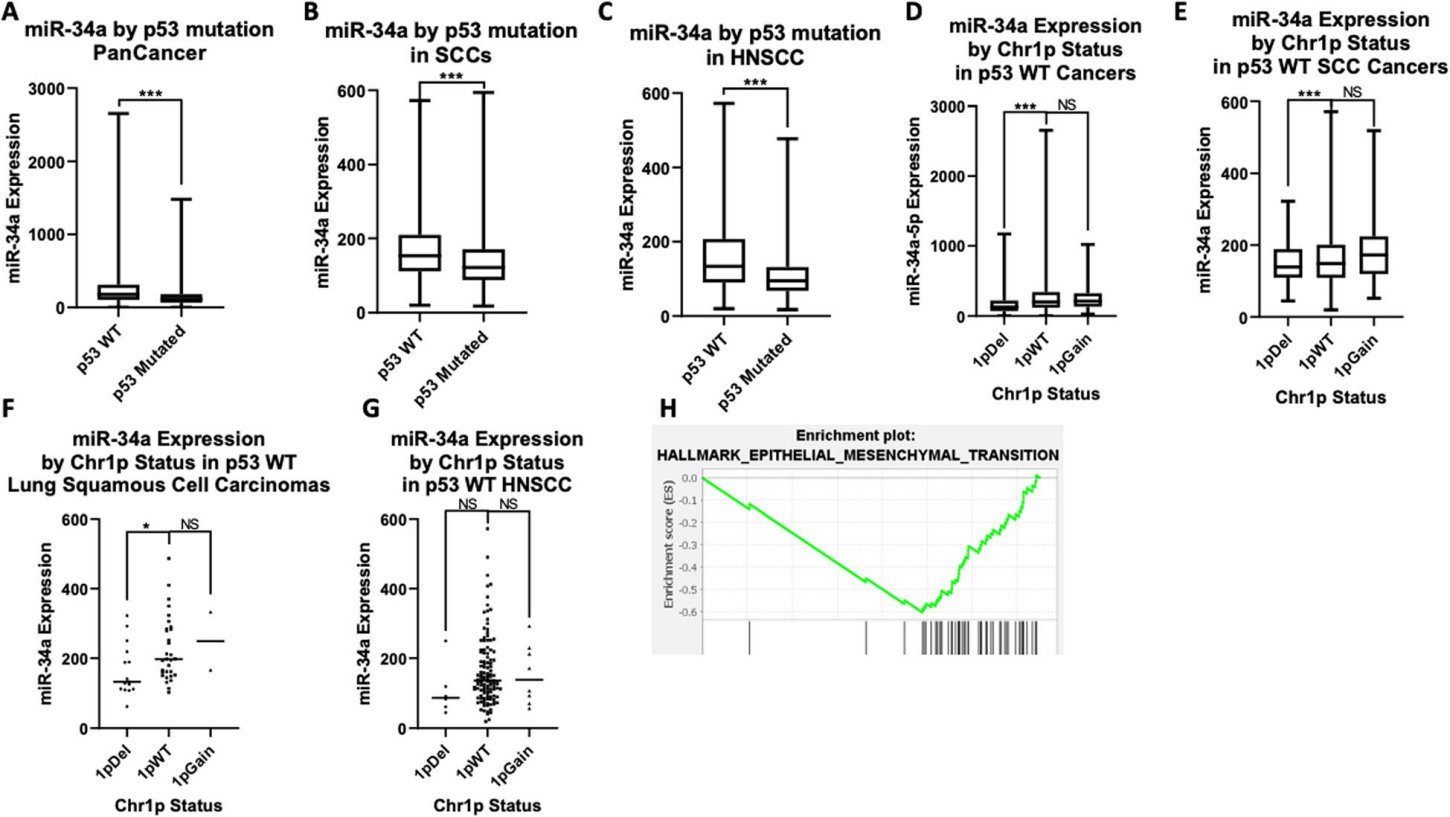
p53 mutation and chr1p deletion correlate with decreased miR-34a expression. **a** through **c**: miR-34a expression versus p53 mutation status in patient tumors across 33 cancer types in the cancer genome atlas (TCGA) pan-cancer dataset (*n* = 9151) (**a**), squamous cell carcinomas (SCCs) (*n* = 1293) (**b**), or head and neck squamous cancers (HNSCCs) (*n* = 490) (**c**). **d** through **g**): miR-34a expression versus chromosome 1p copy number in p53 WT tumors, across cancer (*n* = 7792) (**d**), across SCCs (*n* = 380) (**e**), in lung squamous cell carcinomas (*n* = 57) (**f**), or HNSCCs (*n* = 141) (**g**). **h** Genes whose expression significantly correlated with miR-34a expression were analyzed by gene set enrichment analysis (GSEA) in HNSCC samples (*n* = 497). The plot shows the top enriched hallmark pathway, Epithelial-Mesenchymal Transition (EMT). Y-axis is the GSEA enrichment score. The X-axis is a list of genes ranked by differential expression correlation with miR-34a, with black bars representing genes in the EMT gene set. Data is presented in box and whisker plots, with whiskers representing minimum and maximum. * indicates *p* < 0.05; *** indicates *p* < 0.001

miR-34a is downregulated in different cancers, including breast cancer, lung cancer, bladder cancer, and pancreatic cancer [12, 37, 38]. We analyzed the expression of miR-34a in TCGA pan-cancer tumor samples. As miR-34a is a transcriptional target of P53, we compared miR-34 levels in cases with and without p53 mutation and found significantly lower miR-34a expression in p53 mutated cases compared to p53 wild-type (WT) cases (*P* < 0.001) (Fig. 1a). Similarly, in a squamous cell carcinomas (SCCs) and HNSCC specific analysis, we found a significantly lower expression of miR-34a in cases with p53 mutation (P < 0.001) (Fig. 1b and c). miR-34a is located on chromosome arm 1p, and 1p deletion in non-mutated p53 cases was associated with a lower level of miR-34a in the pan-cancer analysis (*P* < 0.0001) (Fig. 1d). Chromosome 1p deletion was associated with a lower level of miR-34a in SCCs (P < 0.001) (Fig. 1e), lung squamous cell carcinoma (*P* < 0.05) (Fig. 1f), and HNSCC cases (non-significant (N.S)) (Fig. 1g). No statistically significant differences of miR-34a expression were detected between gain of chromosome 1p or WT chromosome 1p (Fig. 1d-g).

We next assessed the gene expression correlations with miR-34a expression in HNSCC patients in the TCGA dataset. For each mRNA, we calculated a Pearson correlation coefficient measuring its correlation with miR-34a expression, with Bonferroni-corrected *p*-values (Supplemental Table 1A). The correlation coefficients with a Bonferroni p-value below 0.01 were assessed for gene set enrichment analysis (GSEA) in the hallmark, and positional gene sets using the GSEA pre-ranked algorithm [39]. The epithelial-mesenchymal transition (EMT) hallmark pathway was the top anti-correlation (Fig. 1h, Supplementary Tables 1B and 1C, Supplementary Fig. 1), including genes such as SNAI2, ZEB1, ZEB2, TWIST2, and TGFB1. The EMT pathway is down-regulated when miR-34a expression is high, consistent with possible role for miR-34a in inhibition of this pathway.

**Fig. 2:**
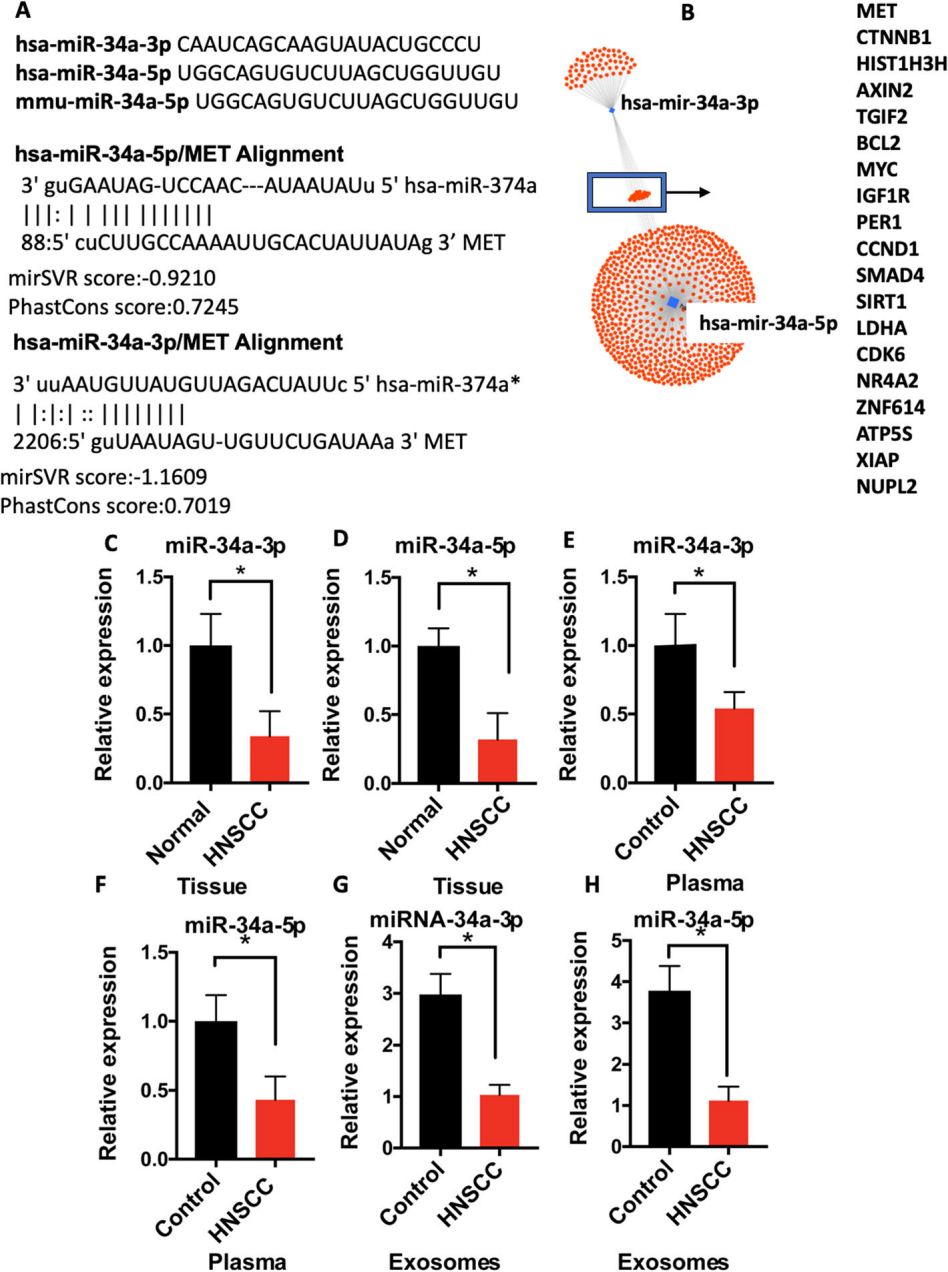
miR-34 is downregulated in head and neck cancer (HNSCC) and targets proto-oncogene MET. **a** Sequences of the mature miR-34a-5p and miR-34a-5p in humans and mice. **b** Bioinformatic analysis suggested direct interaction of both the miR-34a guide strand (hsa-miR-34a-5p) and the passenger strand (hsa-miR-34a-3p) the 3’UTR of MET mRNA. **c** through **h**: Expression of miR-34a-3p and miR-34a-3p were quantified by the TaqMan microRNA Assay in HNSCC tumor samples and matched controls (*n* = 42) (**c** and **d**), HNSCC and healthy plasma (**e** and **f**) and exosomes isolated from HNSCC patients and healthy subjects (*n* = 28) (**g** and **h**). 2^-ΔΔCT^ method was used for relative expression. Data was normalized to RNU48 or spike-in cel-mir-39. Data is presented as mean ± SD. * indicates p < 0.05

The sequences of mature miR-34a-3p (passenger strand) and miR-34a-5p (guide stand) are reported to be conserved among species [40]. Bioinformatics sequence analysis revealed that the MET 3′ UTR has a binding motif for both miR-34a-3p and miR-34a-5p with high stability (mirSVR score of − 0.9210 and PhastCons score of 0.7245 for miR-34a-5p and miRSVR score of − 1.1609 and PHastCons score of 0.7019 for miR-34a-3p) (Fig. 2a, b). mirSVR score ≤ − 0.1 and conserved sites indicated by PhastCOns score > 0.57 were reported as a high predictor of miR-mRNA interaction [41]. We analyzed levels of miR-34a-3p and miR-34a-5p in our cohort of primary HNSCC tumor samples compared to the adjacent tissue (*n* = 42). Levels of both miR-34a-5p and miR-34a-3p were decreased in tumor tissue compared to normal tissue (*P* < 0.05) (Fig. 2c and d). miR-34a levels in plasma isolated from HNSCC patients (*n* = 28) were also lower than in plasma from patients without HNSCC (P < 0.05) (n = 28; Fig. 2e and f). Moreover, levels of miR-34a-5p and miR-34a-3p were significantly lower in the circulating exosomes of patients with HNSCC compared to cancer-free patients (P < 0.05) (Fig. 2g and h).

Checking for absolute quantification of miR-34a-3p and miR-34a-5p in cancerous and healthy tissue, we found about a 50% reduction in miR-34a-5p and miR-34a-3p copy numbers in cancer tissue compared to the normal keratinocytes and normal tissue (Supplementary Fig. 2). However, the miR-34a-3p copy number was overall much lower than miR-34a-5p, which could be attributed to lower stability of the passenger strand in miR biogenesis [42].

**Fig. 3:**
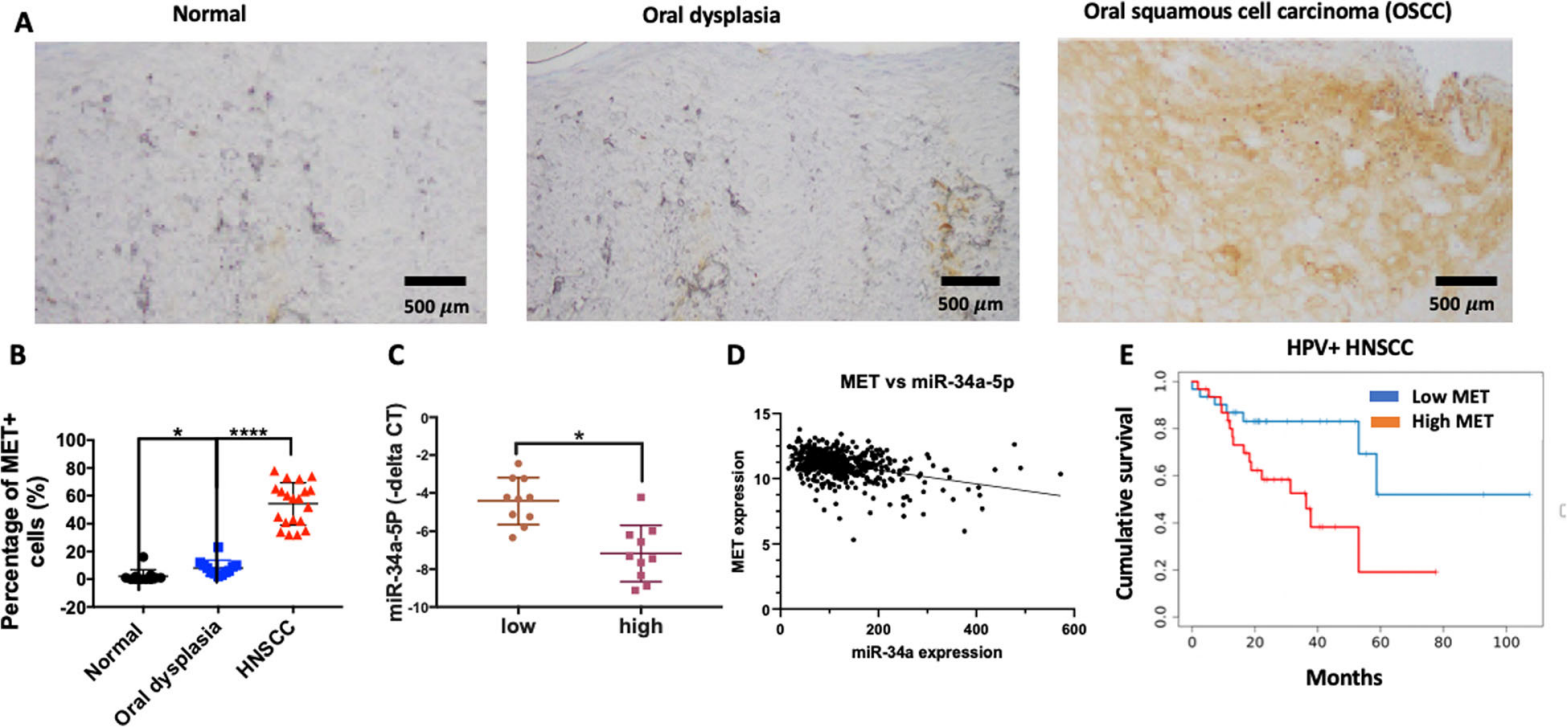
Levels of miR-34a negatively correlate with MET, and MET is overexpressed in head and neck squamous cancer (HNSCC). **a** Immunohistochemistry of MET expression in normal tissue, dysplastic tissue, and HNSCC tissue. **b** The percentage of MET positive cells were quantified in normal tissue, oral dysplasia, and HNSCC. **c** Levels of miR-34a-5p levels were quantified in high MET mRNA HNSCC cases versus low MET mRNA HNSCC cases. **d** Correlation of MET expression and miR-34a levels in over 497 HNSCC cancers in the TCGA dataset (R = -0.38, *p* < 0.0001) (**e**) Cumulative survival of HPV+ HNSCC cases with high MET mRNA compared to low MET mRNA in TCGA database (n = 497). Data represent results of at least three independent experiments. * indicates *p* < 0.05. **** indicates *P* < 0.0001

As our bioinformatics prediction suggested direct targeting of MET by miR-34a-5p, we investigated MET expression in HNSCC samples. In immunohistochemistry of an oral squamous cell carcinoma, MET was highly expressed in oral squamous cell carcinoma/HNSCC cases, but not in oral dysplasia and normal tissue (Fig. 3a). Quantitative scoring of immunohistochemistry showed that the percentage of MET positive cells are significantly increased in oral squamous cell carcinoma (HPV^−^) compared to oral dysplasia and normal tissue (Fig. 3b). We also found an inverse association between MET expression and miR-34a-5p in HNSCC tumors (Fig. 3c). Consistently, we found a significant negative correlation between the expression of MET and miR-34a-5p in HNSCC cases of TCGA data (both HPV+ and HPV-) (Bonferroni corrected *P* < 0.0001) (Fig. 3d). These correlations were likely not due to MET copy number variation (CNV); CNV for MET in HNSCC was so rare that only 4 out of 497 HNSCC samples in TCGA had high MET amplification. In addition, analysis of HNSCC samples with no MET copy number alteration still showed a significant negative correlation between miR-34a expression and MET expression (P < 0.0001) (Supplemental Fig. 3). High expression of MET was associated with significantly lower survival in HNSCC cases, controlling for age, gender, race, and stage (HR: 1.65, 95% CI: 1:1–1.47), *p* = 0.037) (Supplementary Table 2). Cumulative survival analysis showed that HPV^+^ cases with high expression of MET (upper quartile) have significantly less cumulative survival (HR: 1.83; *p* = 0.01) compared to lower quartile (Fig. 3e). There was no correlation between MET expression and survival of HPV^−^ tumors in cumulative survival analysis.

**Fig. 4:**
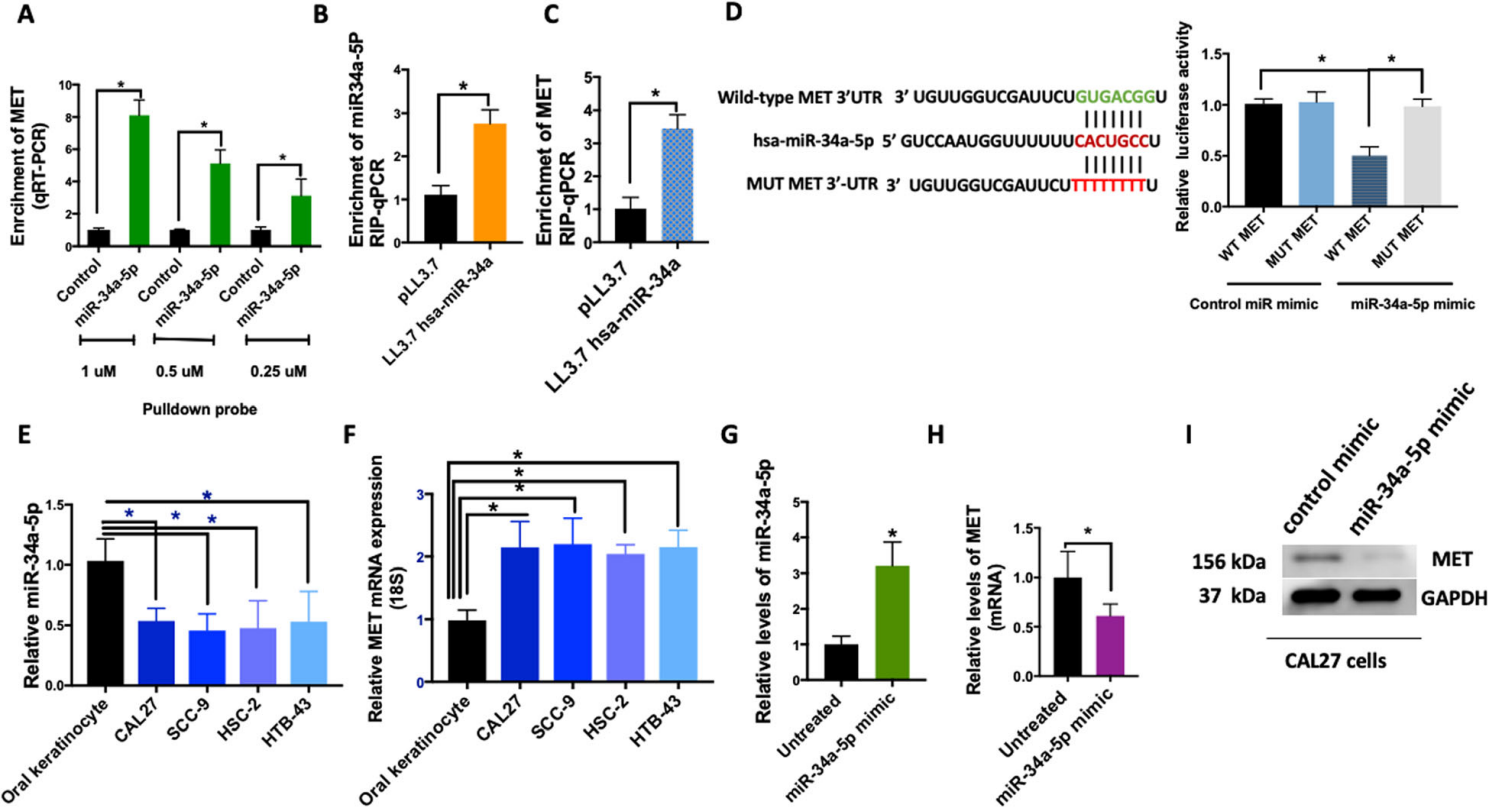
miR-34a directly interacts with MET mRNA and downregulates MET. **a** RNA was isolated from cells, biotin-based pull-down probe for miR-34a-5p or negative control probe at different concentrations to assess interaction of miR-34a-5p with MET in CAL27 cells was used. The enrichment of MET was quantified by qRT-PCR. **b** and **c** The miR-34a-5p overexpressing CAL27 cells were subjected to an anti-Ago2 CHIP assay. Levels of MET and miR-34a-5p were quantified by RIP-qPCR. **d** Dual-luciferase assay was performed in cells transfected with miR-34a-5p mimic or a control miR mimic and a luciferase reporter construct encoding the luciferase gene fused either to a mutated MET 3′ UTR (MUT) or a wild-type MET 3′ UTR (WT). **e** and **f** MET expression and miR-34a-5p expression in HNSCC cell lines were quantified by a TaqMan microRNA assay. **g** miR-34a-5p mimic was introduced to CAL27 cells by electroporation (25 nM). Levels of miR-34a-5p was quantified by a TaqMan microRNA assay after 12 h. **h** MET mRNA expression was quantified 24 h after over-expression of miR-34a-5p mimic. **i** Western blot measuring MET protein expression with and without expression of miR-34a-5p mimic. All experiments were performed in CAL27 cells. All experiments were performed in triplicate. Data represent results of at least three independent experiments. Data is presented as mean ± SD.* indicates *p* < 0.05

We next studied the interaction of miR-34a-5p with MET in HNSCC cell lines. To investigate the direct interaction of miR-34a-5p with MET mRNA, we performed pulldown experiments with biotinylated miR-34a-5p or negative control probes and quantified levels of mRNA in the pulldown by qRT-PCR. We observed a dose-dependent enrichment of MET mRNA with miR-34a-5p probes suggesting a direct interaction (Fig. 4a). miRs exert their gene expression regulatory function through translational inhibition or transcript degradation via Argonaute 2 (Ago2)-catalyzed cleavage [43]. Thus, we assessed the association of Ago2 with miR-34a-5p and MET to determine whether miR-34a was recruiting AGO2 to MET mRNA. Overexpression of miR-34a-5p by plasmid followed by anti-Ago2 CHIP and RNA-binding protein immunoprecipitation (RIP)-qPCR on the whole cell lysate showed a complex of MET, miR-34a-5p with Ago2 in CAL27 cells. (Fig. 4b&c). Luciferase assay further confirmed the direct interaction of miR-34a seed sequence with the MET 3’UTR, where a significant decrease in luciferase activity was detected when CAL27 cells were co-transfected with the WT 3’UTR of MET and not a mutated 3’UTR (Fig. 4d).

Next, we assessed whether miR-34a-5p level in the HNSCC cell lines. The level of miR-34a-5p was down in all the HNSCC cell lines examined compared to oral keratinocytes (*P* < 0.001) (Fig. 4e), and the mRNA levels of MET were elevated in those cell lines (P < 0.001) (Fig. 4f). As a more direct test to assess the effect of higher miR-34a on MET levels, we used a miR-34a-5p mimic, a synthetic miR. Introduction of the miR-34a-5p mimic in CAL27 cells resulted in a decrease of MET mRNA and protein levels (Fig. 4g-i).

**Fig. 5:**
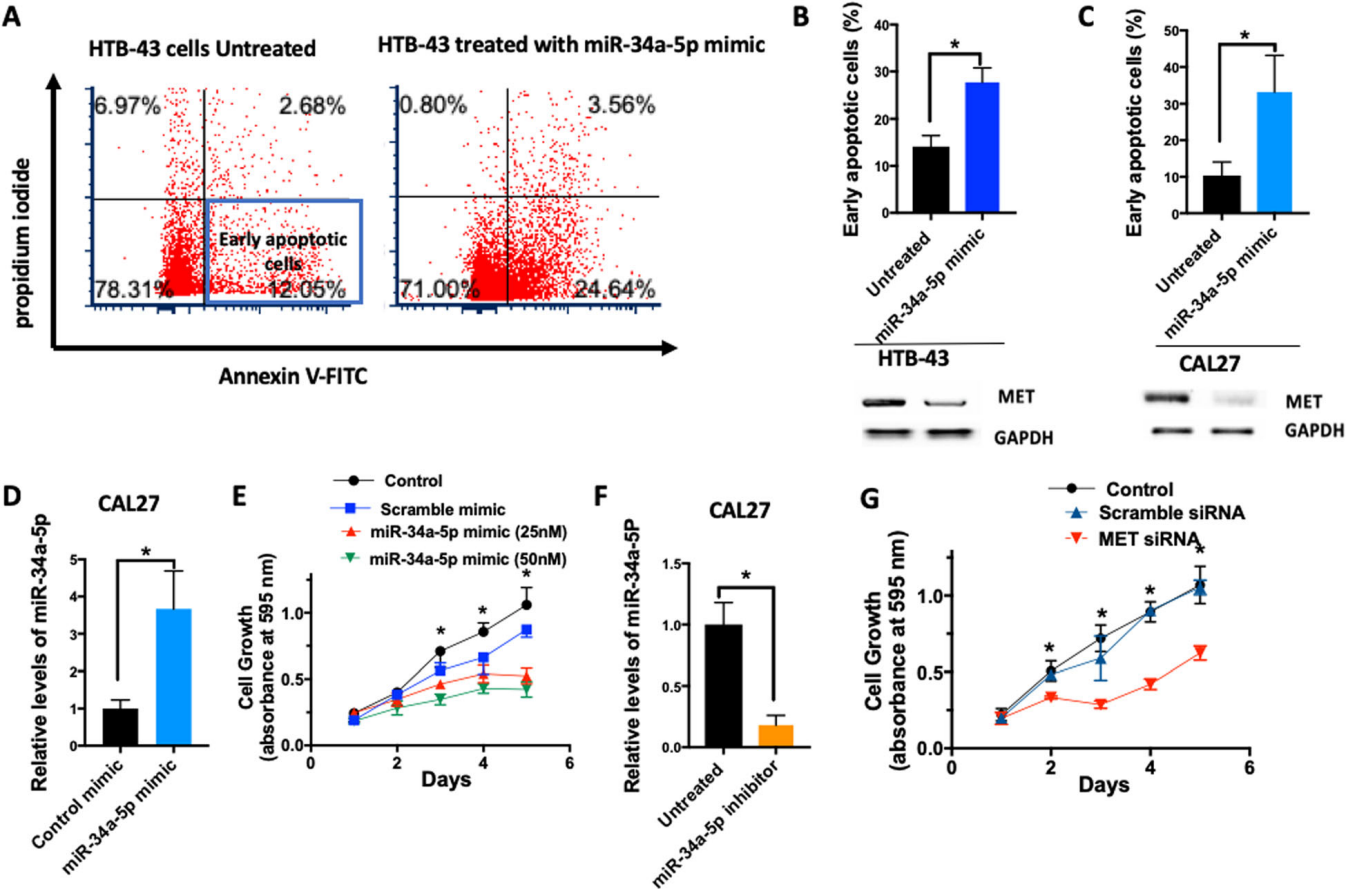
miR-34a over-expression induces early apoptosis, prevents tumor proliferation, and inhibits tumor growth in vivo. **a** Representative flow cytometry plot of annexin V (FITC) versus propidium iodide in control mimic treated cells or miR-34a-5p treated HTB-42 cells (25 nM, 48 h). Early apoptotic cells were defined as propidium iodide low and Annexin-V high. **b**, **c** The early apoptosis detection assay determined the percentage of early apoptotic cells in 2 different head and neck squamous cell lines (HTB-43 and CAL27 cells), and MET expression was confirmed by western blot. **d** The level of miR-34a-5p expression was determined by a TaqMan microRNA assay, 12 h post electroporation of miR-34a mimic or control mimic (25 nM). **e** miR-34a-5p mimic (25 nM or 50 nM) was administered to cells at day 0, and proliferation was measured using MTT reagent to assess the effect of miR-34a on the proliferation of CAL27 cells. **f** and **g** Anti-LNA-miR-34a (miR-34a-5p inhibitor) was administered to CAL27 cells and a TaqMan microRNA Assay was used to quantify levels of miR-34a-5p. MET siRNA (20 nM) or scramble siRNA (20 nM) were introduced to CAL27 cells which received miR-34a-5p inhibitor by Lipofectamine RNAiMAX and cell proliferation was quantified by MTT assay. All experiments performed at least in triplicate. Data represent results of at least three independent experiments. Data is presented as mean ± SD.* indicates p < 0.05

miR-34a has been suggested to play a significant role in cancer cell proliferation, apoptosis, and migration [43]. Thus, we further investigated the role of miR-34a in head and neck carcinogenesis using the miR-34a-5p mimic. Upon administration of the mimic in two different HNSCC cell lines (HTB-43, CAL27), measurement of annexin V and propidium iodide staining demonstrated an increase in the percentage of early apoptotic cells as well as suppression of MET expression (Fig. 5a-c). Introduction of the miR-34a-5p mimic led to dose-dependent inhibition of proliferation in the CAL27 cell line (*P* < 0.05) (Fig. 5d, e) and HCSS-4 cell line (Supplementary Fig. 4). To assess whether the anti-apoptotic effects of miR-34a-5p are at least partially exerted via MET signaling, we inhibited miR-34a in CAL27 cells using anti-miR technology (Fig. 5f) and treated cells with MET siRNA. Suppression of MET inhibited proliferation (Fig. 5g).

**Fig. 6:**
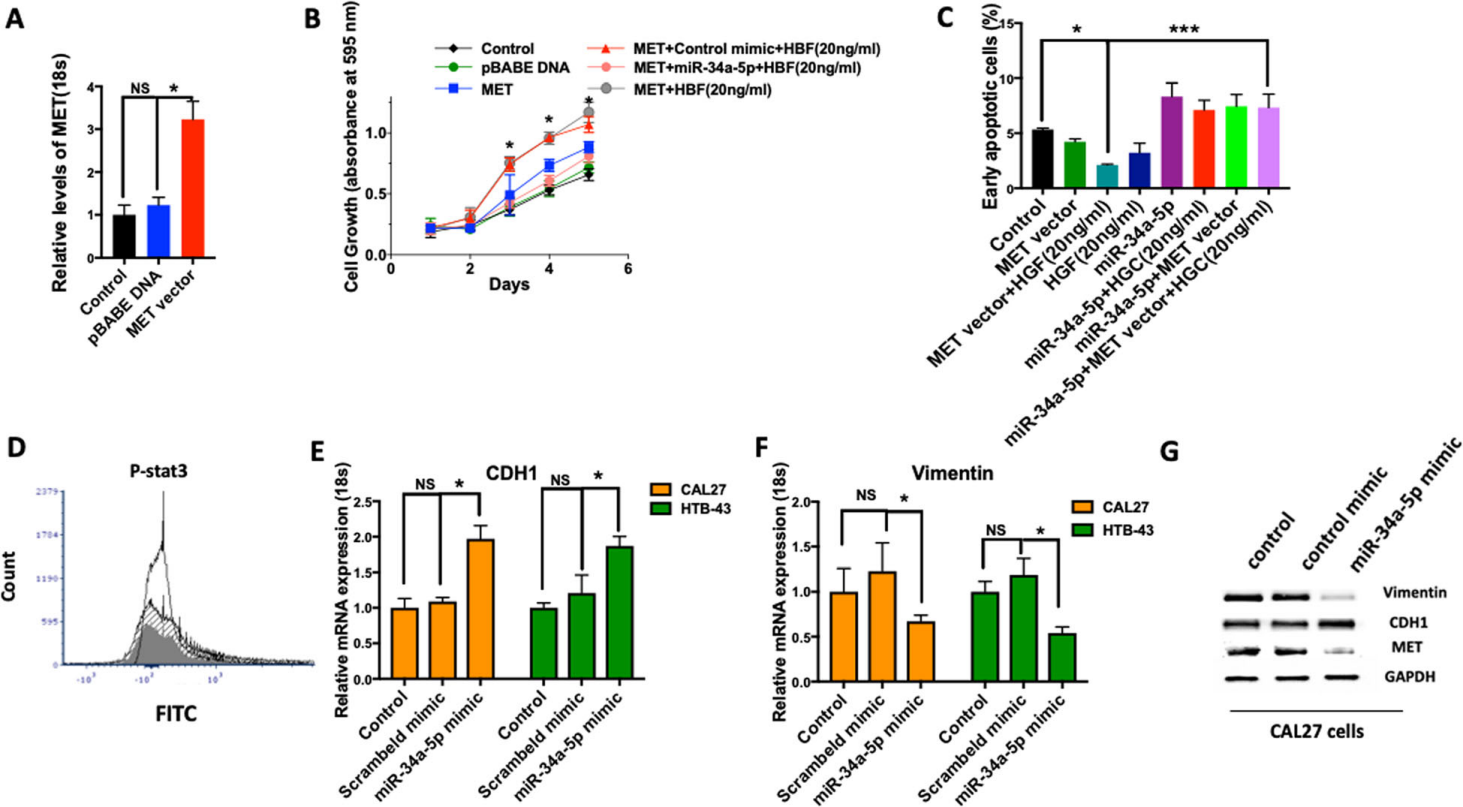
The anti-tumorigenic effect of miR-34a is dependent on MET, and miR-34a-5p inhibits epithelial mesenchymal transition (EMT). **a** MET vector or control vector (pBABE) were introduced to CAL27 cells and levels of MET mRNA expression were quantified by RT-qPCR. **b** miR-34a-5p (25 nM) mimic was administrated to the MET overexpressing CAL27 cells and cell proliferation was measured after 24 h using MTT assay. MET signaling was stimulated with HGF (20 ng/ml). **c** Percentage of early apoptotic cells were determined by Annexin V and PI in MET overexpressing CAL27 cells with and without introduction of miR-34a-5p mimic. MET signaling was stimulated with HGF (20 ng/ml). **d** Effect of miR-34a-5p on STAT3 protein phosphorylation (shaded line: control; open line: HGF (20 ng/ml); gray line: HGF (20 ng/ml) + miR34a-5p mimic). **e**, **f** Relative mRNA expression of epithelial marker (CDH1) and mesenchymal marker (Vimentin) were quantified 48 h after introduction of the miR control mimic or miR-34a-5p mimic by Lipofectamine RNAiMAX in CAL27 cells. RT- qPCR data was normalized using 18 s. **g** Protein expression of CDH1 and Vimentin were determined after 48 h administration of miR-34a-5p mimic or control mimic in CAL27 cells using western blot. Data represent results of at least three independent experiments. Data is presented as mean ± SD. * indicates *p* < 0.05. *** < 0.001

To determine whether any of the effects of MET overexpression are dependent on miR-34a-5p, we generated CAL27 cells with MET overexpression (Fig. 6a). MET overexpression-induced cell growth and proliferation was attenuated by the miR-34a-5p transient introduction (Fig. 6b). In addition, our rescue experiment demonstrated that miR-34a-5p can reverse the anti-apoptotic effect of MET overexpression (Fig. 6c).

As it has previously been shown that activated MET induces tumor invasion via phosphorylation of STAT3, we assessed levels of phospho-STAT3 (p-STAT3) and found decreased p-STAT3 after the introduction of miR-34a-5p mimic in CAL27 cell lines (Fig. 6d). As our bioinformatics analysis in TCGA tumors showed negative correlations of miR-34a-5p with the expression of epithelial-to-mesenchymal transcription factors such as SNAI2, ZEB1, ZEB2, and TWIST2 and presented in Fig. 1g, we assessed expression changes of epithelial and mesenchymal markers upon miR-34a-5p overexpression in HNSCC cell lines. Interestingly, the administration of miR-34a-5p led to a decreased mRNA expression of the mesenchymal marker vimentin and increased expression of epithelial marker CDH1 in CAL27 and HTB-43 (Fig. 6e & f), consistent with the inhibition of EMT. Similarly, the administration of miR-34a-5p led to a decreased in Vimentin protein expression and an increase in CDH1 protein expression.

**Fig. 7:**
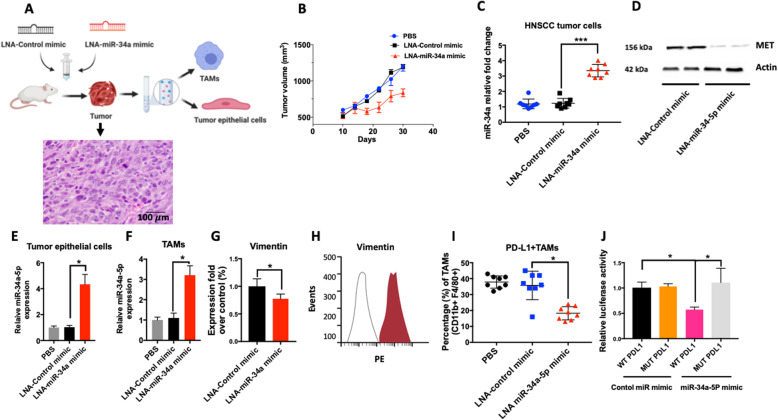
Administration of miR-34a-5p mimic inhibit tumors growth and PDL1 expressing tumor associated macrophages (TAMs). **a** 2 × 10^6^ HTB-43 cells were injected to the tongue of nude mice. LNA-control mimic or LNA-miR-34a mimic were once a week (20 mg/kg; intraperitoneal) for two weeks, starting 10 days after tumor cell injections. Mice were euthanized after 20 days and epithelial cell and TAMs were isolated after tissue dissociation using a tumor dissociation kit and isolation of cells with magnetic beads (CD326 (EpCAM) for tumor epithelial cells and CD11b for TAMs). A representative histology of HTB-43 xenograft tumor is included. **b** Tumor volume was measured by caliper (*N* = 8 per group). **c** Level of miR-34a-5p was quantified in tumor lysate using a TaqMan microRNA assay (N = 8 per group). **d** Levels of MET were assessed in tumor lysates of mice that received the LNA control mimic or the LNA-miR-34a mimic. **e**, **f** Levels of miR-34a-5p was quantified in isolated tumor epithelial cells after isolation by Anti-EpCAM magnetic beads (*n* = 8 per group) and TAMs after isolation by Anti-CD11b magnetic beads (n = 8 per group). **g** The percentage fold over control of Vimentin expression in mice that received the LNA-control miR or LNA-miR-34a-5p mimic (*n* = 6 mice per group) measured by qRT-PCR. **h** Levels of Vimentin protein expression were quantified in tumor cells with rat anti-mouse vimentin APC-conjugated antibody. Filled histogram represents the mice that received LNA-control miR, and open histogram represents the mice that received LNA-miR-34a-mimic. **i** Percentage of PDL1 expressing TAMs (defined as CD45^+^CD11b^+^F4/80^+^ cells) were quantified by flow cytometry (n = 8 per group). **j** Luciferase activity in cells transfected with miR-34a-5p mimic or a control miR mimic and a luciferase reporter construct encoding the luciferase gene fused either to the a mutated PDL1 3′ UTR (MUT) or a wild-type PDL1 3′ UTR (WT) in RAW 264.7 cells. Data represent results of at least three independent experiments. * indicates p < 0.05. Data is presented as mean ± SD. * indicates p < 0.05; *** indicates *p* < 0.001

In order to determine the efficacy of restoring miR-34a-5p level as a therapeutic strategy, we used an LNA-miR-34a-5p mimic. LNA-miR-34a-5p was injected intraperitoneally (i.p) into an orthotopic xenograft mouse model of oral cancer (Fig. 7a). Tumor growth was significantly attenuated in the mice that received the LNA-miR-34a-5p mimic compared to those that received the control mimic or vehicle (Fig. 7b). The level of miR-34a-5p was significantly increased in the tumors of mice that received LNA-miR-34a-5p (Fig. 7c). The level of MET was significantly deceased in the lysate of tumors isolated from these mice as well (Fig. 7d). Isolation of tumor epithelial cells and TAMs showed significant increases in the level of miR-34a-5p in both cell types after administration of LNA-miR-34a-5p mimic (Fig. 7e, f). The mesenchymal marker vimentin was significantly decreased in the treatment group, indicating suppression of EMT by miR-34a-5p (Fig. 7g, h). Flow cytometry analysis on TAMs showed a decrease in the PDL1^+^TAMs (CD11b + F4/80+) in the group that received LNA-mir-34a-5p treatment (Fig. 7i). PDL1 is reported to be direct target of miR-34a-5p [44]. Consistently, a luciferase assay confirmed the direct interaction of miR-34a-5p seed sequence with the PDL1 3’UTR, where a significant decrease in luciferase activity was detected when RAW macrophages were co-transfected with the WT 3’UTR of PDL1 (Fig. 7j). Taken together, these data support the mechanistic connection of miR-34a-5p in suppressing oncogenic MET and restoring tumor immunity through targeting PDL1.

As the tumor microenvironment is composed of different cell types and miR-34a plays a role in tissue homeostasis, we evaluated the correlation between miR-34a expression and cellular subtypes in TCGA data. The cellular subtype estimates were generated by xCell. Among the top hits, higher expression of miR-34a was correlated with a significantly higher level of pro-B cells [p (FDR) = 4.42E-13], CD8 naïve T-cells [p (FDR) = 0.008245842] and Th1 cells [p (FDR) = 3.83E-7] in HNSCC tumors (Supplementary Table 3).

## Discussion

MicroRNAs play an important role in the progression of HNSCC tumorigenesis and growth [27, 28, 45–47]. It has been suggested that specific miRs could act as a molecular diagnostic tool for HNSCC [48, 49], and miR-based therapy could potentially be a rational approach for the therapeutic targeting of HNSCC [50]. Identifying novel targets that would be efficient in HNSCC therapy is critical. In the present study, for the first time, we investigated the role of the miR-34a-MET axis in the pathogenesis of HNSCC. Our analysis of TCGA data demonstrated that downregulation of miR-34a is associated with p53 mutation and chromosome arm 1p deletion in HNSCC and lung squamous cell carcinoma. Additionally, higher levels of miR-34a-5p were correlated with lower levels of key transcription factors of EMT. We found significantly decreased levels of miR-34a-5p in tumor tissue and circulation of patients with HNSCC. We found that miR-34a-5p acts as a tumor suppressor, directly represses the proto-oncogene MET, and modulates cell proliferation. Lastly, our work demonstrates the role of miR-34a in maintaining tumor immunity, as administration of LNA-miR-34a-5p mimic resulted in a decrease in the percentage of PDL1^+^TAMs, which are reported to have pro-tumorigenic and immunosuppressive activity [51, 52].

miR-34a is down-regulated in many cancers [43, 53–55], and there are several hypotheses that explain this down-regulation [44, 56–58]. By leveraging the TCGA dataset, we demonstrated two possible mechanisms of miR-34a-5p down-regulation in cancer. Consistent with previous work, p53 mutation correlates with decreased expression of miR-34a, suggesting miR-34a as a transcriptional target of p53. In addition, miR-34a is located on chromosome 1p, a chromosome arm that is frequently deleted in cancer [13]. We also demonstrated that chromosome 1p deletion correlates with decreased miR-34a expression. This is not surprising, as DNA copy number generally correlates with gene expression, but also provides another explanation for decreased miR-34a in HNSCC. In addition, these findings suggest that chromosome arm-level deletion could serve as a biomarker for response to miR-34a-based therapeutic approaches. We are actively pursuing aneuploidy as a biomarker for drug response, including for miR-34a and other miRs. Interestingly, the effect of copy number on miR-34a expression is masked by p53 mutation; if p53 is mutated, there is no correlation between chromosome 1p copy number and miR-34a expression. These data are consistent with *trans* transcriptional regulation serving a dominant role over *cis* DNA levels.

Our mechanistic experiments showed that miR-34a directly interacts with the proto-oncogene MET and attenuates the MET signaling axis by posttranscriptional gene regulation in cancer cells. Activating point mutations of MET and MET amplification have been reported in several cancer types, including gastric cancer [59], breast cancer [60], hepatocellular carcinoma [61, 62], and non-small cell lung cancer [63, 64]. MET plays an important role in the occurrence, development, invasion, and metastasis of malignant tumors [65–67]. While MET is a validated drug target in lung cancer, the best biomarker strategy for enrichment of a susceptible patient population still remained undefined [63]. Activation of HGF/MET signaling correlates with increased recurrence rates and poor prognosis in HNSCC [68, 69]. MET is a potential novel therapeutic target for HNSCC and a greater-than-additive inhibition of cell growth was observed when combining a MET inhibitor with cisplatin or erlotinib [70].

In preclinical HNSCC models, MET expression and activation have also been shown to be associated with resistance to anti-EGFR therapeutics. Novoplansky et al. reported an association between MET amplification and overexpression with HNSCC progression, and potentially overactive MET played a causative role in the development of resistance to cetuximab in HNSCC [71]. Interestingly, MET induces MAPK re-activation in all tested HNSCC models, and blocking of MAPK with a MET inhibitor re-sensitized the HGF-stimulated tumor cells to cetuximab [71]. Similarly, MET knock down sensitized two cetuximab resistant non-small cell lung adenocarcinoma cell lines, LXFA 526 L and LXFA 1647 L, to EGFR inhibition [69]. We found high expression of MET throughout the entire epithelium in oral cancer compared to partial staining in oral dysplastic lesions and no expression in normal tissue. The mechanistic role of MET in the transformation of pre-cancerous lesions to cancerous lesions should be further investigated in future studies.

Direct interaction of mir-34a-5p with PDL1 has been reported previously [44]. The role of the PDL1- PD1 axis in facilitating tumor escape from immune control has led to an active therapeutic target in multiple cancer types [72, 73]. However, the fact that PDL1 is expressed not only in cancer cells but also in immune cells with the highest abundance in TAMs has only been recently reported [74–76]. TAMs are one of the major cell populations in the tumor microenvironment [77], and they express the vast majority of PDL1 in tumors. The level of PDL1 expression in TAMs could determine the efficacy of PDL1 pathway blockade [51, 74]. TAMs that express PDL1 can suppress T cells in the tumor microenvironment and contribute to tumor immune evasion [78, 79].

The pleiotropic nature of miRs and their involvement in all cancer hallmarks make them particularly attractive drug targets for cancer treatment. A challenge of miR-based therapy is the delivery of miRs in the right dose to the target tissue. Different drug delivery vehicles have been proposed for miR delivery, including liposomes and exosomes. Liposomes are immunogenic, subject to rapid clearance by the immune system after administration [80]. In contrast, exosomes, naturally occurring nanovesicles, are not immunogenic and can be used effectively for miR-based therapy [81]. Another strategy is the chemical modification of miR-mimics or inhibitors to induce higher stability and cell penetration. In accessible solid tumors, intra-tumoral injections of miR-based therapies into the pathogenic site could improve efficacy and minimize side effects [82]. In the present study, we showed that restoration of miR-34a-5p using LNA-miR34-5p could inhibit tumor growth and progression and restore anti-tumor immune function in HNSCC.

## Conclusions

This study presents strong evidence that miR-34a-5p acts as a tumor suppressor and physically interacts with and functionally targets the proto-oncogene MET. miR-34a-5p overexpression may have a potential therapeutic benefit in HNSCC via MET inhibition and restoration of anti-tumor immunity. In particular, miR-34a-5p overexpression might be useful as adjuvant therapy or monotherapy in HNSCC.

## Supplementary Information

**Additional file 1: SF1**- Expression of hallmark epithelial-mesenchymal transition genes anti-correlates with miR-34a expression in HNSCC. Each column represents a different tumor sample (*n* = 499), and each row a different hallmark EMT gene. Tumors are sorted by expression of miR-34a (top row). Higher expression is in red, lower expression in blue. Values for each gene per tumor are included in Supplemental Table 1C.

**Additional file 2: SF2**- miR-34a-5p is more abundant in tumor and normal tissue compared to the miR-34a-3p. The absolute number of miR-34a-5p and has-miR-34a-3p was quantified by qPCR after construction of standard curve by spike-in cel-miR-39.

**Additional file 3: SF3**-MET expression anti-correlates with miR-34a expression in TCGA HNSCC. HNSCC samples without any shallow or deep MET copy number alteration were plotted for MET expression vs. miR-34a expression (*n* = 304). *P* < 0.0001.

**Additional file 4: SF4**- miR-34a overexpression induces tumor cell proliferation. The miR-34a-5p mimic or control miR mimic were administered to HCSS-4 cells, and proliferation was measured after 24 h using MTT reagent to assess the effect of miR-34a-5p on cellular proliferation.

**Additional file 5: Supplemental Table 1.** A. Gene expression correlations with miR-34a expression. B. GSEA gene sets (hallmark and positional) with FWER < 0.05. C. Gene expression of hallmark EMT genes by miR-34a expression.

**Additional file 6: Supplemental Table 2.** Cox Proportional hazard model to assess overall survival based on MET expression in head and neck cancer patients in TCGA data.

**Additional file 7: Supplemental Table 3.** Immune Infiltrate Correlations. A. Positive Correlation with miR-34a expression. B. Negative Correlation with miR-34a expression.

## Data Availability

The datasets supporting the conclusions of this article are included within the article (and its additional files).

## Abbreviations

AGO2: Argonaute 2
CNV: Copy Number Variation
DMEM: Dulbecco’s Minimal Essential Medium
EMEM: Eagle’s Minimum Essential Medium
EMT: Epithelial-to-Mesenchymal Transition
FDR: False Discovery Rate
GSEA: Gene Set Enrichment Analysis
HNSCC: Head and Neck Squamous Cell Carcinoma
HPV: Human Papillomavirus
HR: Hazard Ratio
IP: Immunoprecipitation
LNA: Locked Nucleic Acid
miR: microRNA
PDL1: Programmed Death Ligand 1
SEM: Standard error of mean
TCGA: The Cancer Genomic Atlas
TAMs: Tumor Associated Macrophages
UTR: Untranslated region
qRT-PCR: Quantitative Reverse Transcription Polymerase Chain Reaction
RIP-PCR: RNA Immunoprecipitation- Polymerase Chain Reaction
