# Supplementary figures and images for: Down-regulation of the tumor suppressor miR-34a contributes to head and neck cancer by up-regulating the MET oncogene and modulating tumor immune evasion
Source: J Exp Clin Cancer Res. 2021 Feb 17;40:70. doi: 10.1186/s13046-021-01865-2 (PMC7890893; doi:10.1186/s13046-021-01865-2)

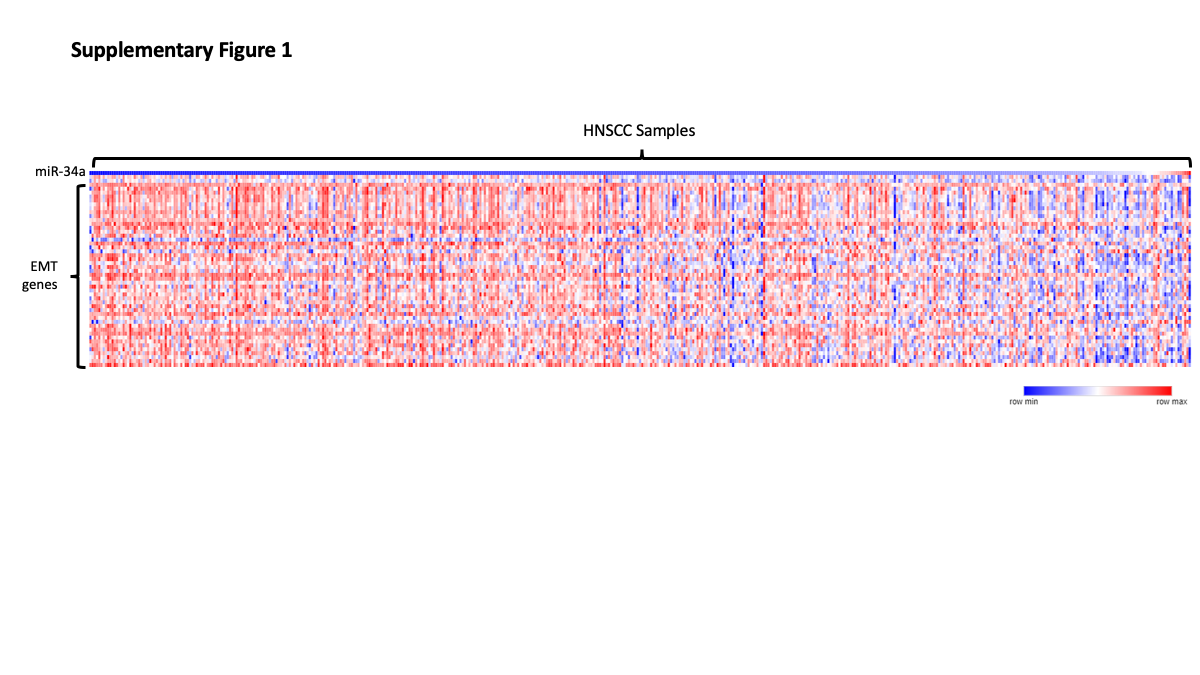

Supplement: Supplementary file 1 — Additional file 1: SF1- Expression of hallmark epithelial-mesenchymal transition genes anti-correlates with miR-34a expression in HNSCC. Each column represents a different tumor sample (n = 499), and each row a different hallmark EMT gene. Tumors are sorted by expression of miR-34a (top row). Higher expression is in red, lower expression in blue. Values for each gene per tumor are included in Supplemental Table 1C. [file 13046_2021_1865_MOESM1_ESM.tiff]

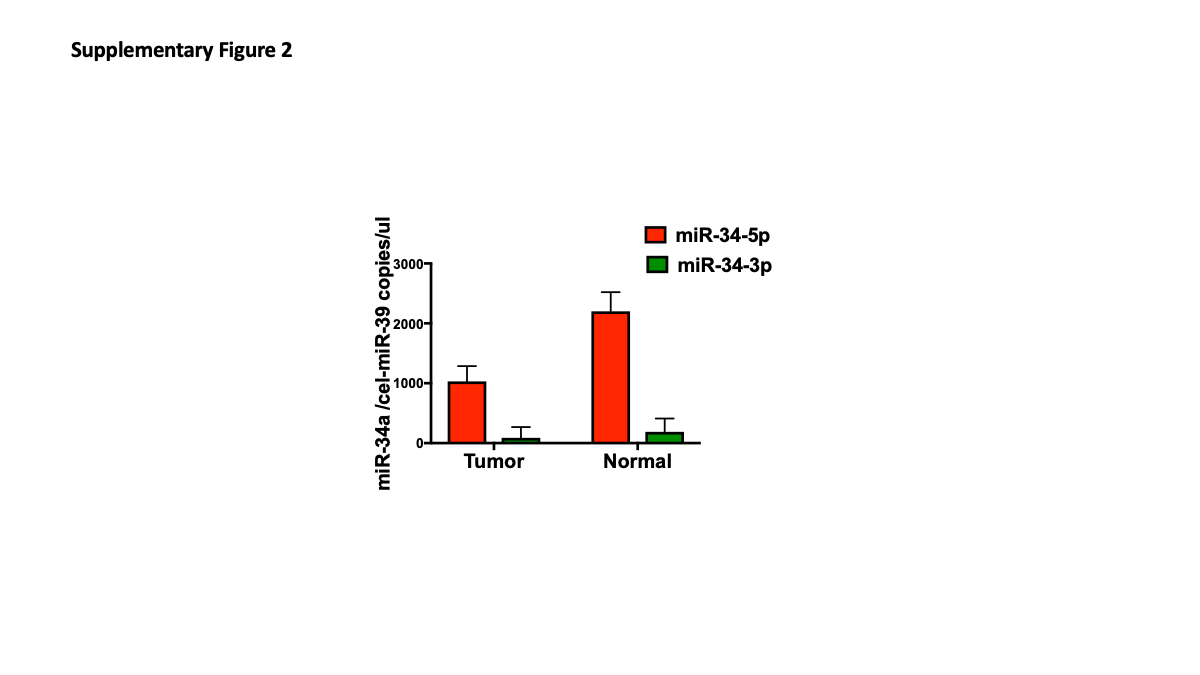

Supplement: Supplementary file 2 — Additional file 2: SF2- miR-34a-5p is more abundant in tumor and normal tissue compared to the miR-34a-3p. The absolute number of miR-34a-5p and has-miR-34a-3p was quantified by qPCR after construction of standard curve by spike-in cel-miR-39. [file 13046_2021_1865_MOESM2_ESM.tiff]

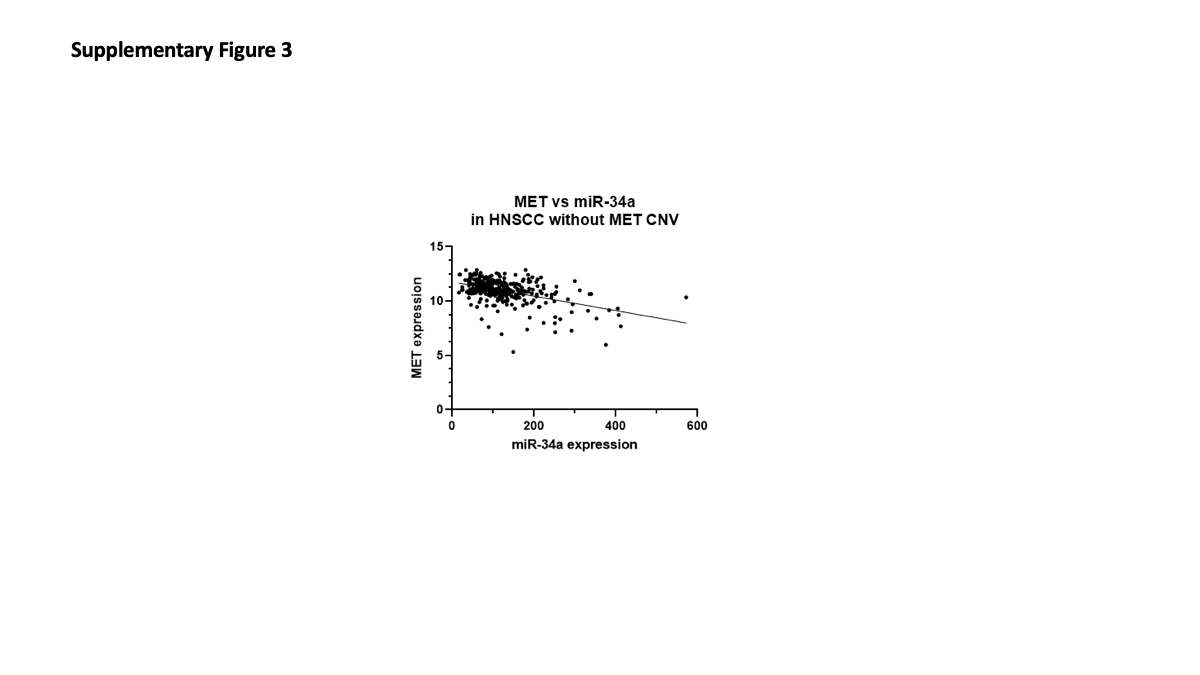

Supplement: Supplementary file 3 — Additional file 3: SF3-MET expression anti-correlates with miR-34a expression in TCGA HNSCC. HNSCC samples without any shallow or deep MET copy number alteration were plotted for MET expression vs. miR-34a expression (n = 304). P < 0.0001. [file 13046_2021_1865_MOESM3_ESM.tiff]

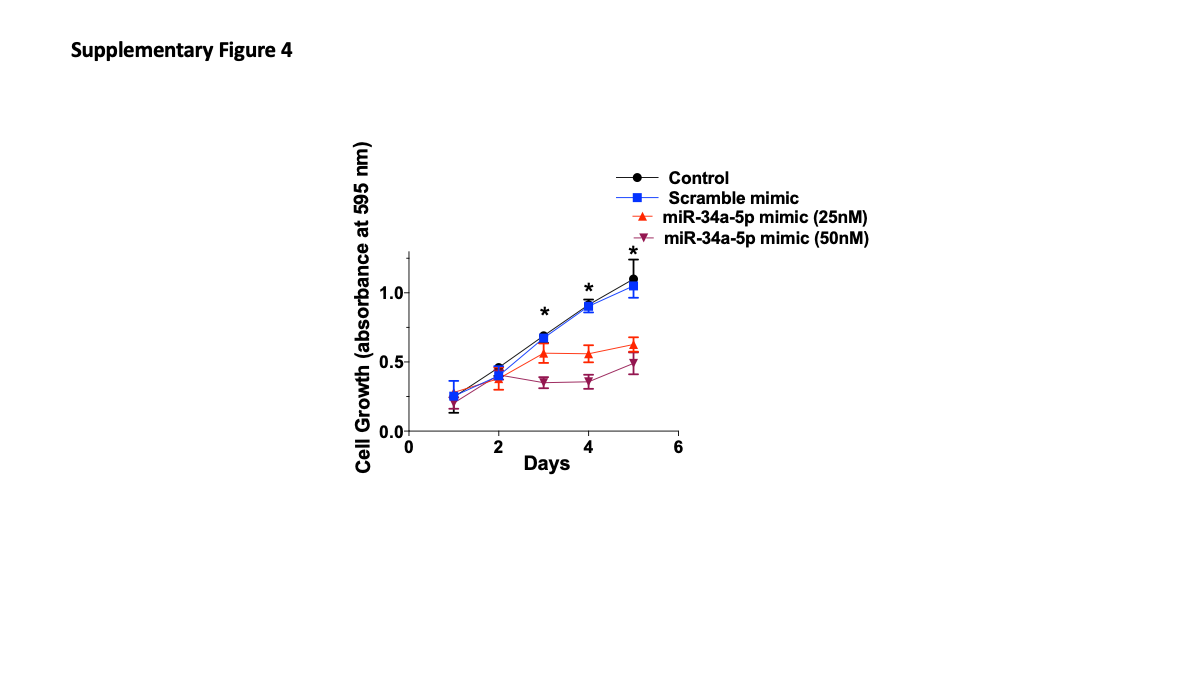

Supplement: Supplementary file 4 — Additional file 4: SF4- miR-34a overexpression induces tumor cell proliferation. The miR-34a-5p mimic or control miR mimic were administered to HCSS-4 cells, and proliferation was measured after 24 h using MTT reagent to assess the effect of miR-34a-5p on cellular proliferation. [file 13046_2021_1865_MOESM4_ESM.tiff]
