# Supplementary material for: Down-regulation of the tumor suppressor miR-34a contributes to head and neck cancer by up-regulating the MET oncogene and modulating tumor immune evasion
Source: J Exp Clin Cancer Res. 2021 Feb 17;40:70. doi: 10.1186/s13046-021-01865-2 (PMC7890893; doi:10.1186/s13046-021-01865-2)
Supplement: Supplementary file 6 — Additional file 6: Supplemental Table 2. Cox Proportional hazard model to assess overall survival based on MET expression in head and neck cancer patients in TCGA data. [file 13046_2021_1865_MOESM6_ESM.docx]

Supplementary Table 2- Cox Proportional hazard model to assess overall survival based on MET expression in head and neck cancer patients in TCGA data

|  | coefficient | HR | 95%CI_lower | 95%CI_upper | p |
| --- | --- | --- | --- | --- | --- |
| MET | 0.156 | 1.169 | 1.010 | 1.354 | 0.037 * |
| Age | 0.020 | 1.020 | 1.006 | 1.035 | 0.006 ** |
| Gender (male) | -0.267 | 0.765 | 0.554 | 1.058 | 0.106 |
| Race (Black) | 0.110 | 1.116 | 0.374 | 3.327 | 0.844 |
| Stage2 | 0.666 | 1.946 | 0.672 | 5.638 | 0.220 |
| Stage3 | 0.934 | 2.546 | 0.890 | 7.285 | 0.082 |
| Stage4 | 1.323 | 3.755 | 1.384 | 10.188 | 0.009 ** |

*MET in HNSC (n=522; 431 patients with 183 dying); *p<0.05; **p<0.01; Wald test p= 1.22e-04; Likelihood ratio test p = 3.41e-05*
